# P08-12 Equity review of the relationship between the physical environment and physical activity

**DOI:** 10.1093/eurpub/ckac095.125

**Published:** 2022-08-29

**Authors:** Lars B Christiansen, Jens Høyer-Kruse, Marlene R. L Pedersen, Mette B Eriksen, Anne F Hansen

**Affiliations:** 1 Department of Sports Science and Clinical Biomechanics, University of Southern Denmark, Odense, Denmark; 2 University Library Research and Analytics, University of Southern Denmark, Odense, Denmark

**Keywords:** review, sociodemographic, moderators, population, cities

## Abstract

**Background:**

The impact of the physical environment on physical activity is not equal for all citizens. According to the socioecological theory, moderators of the association can for example be income, education, age, ethnicity and gender. These moderating conditions are important for practice in order to plan and initiate the best solutions for different population groups.

**Methods:**

Based on a systematic review process 1464 studies were screened. After title, abstract and full text reading 41 studies remained and constitute the basis for this presentation.

**Results:**

Citizens with different sociodemographic backgrounds are often geographically divided within cities. Looking at the overall characteristics of urban areas, studies show that citizens with lower incomes and shorter education often live in areas with higher population density and generally shorter distances to daily destinations. Conversely, citizens with higher incomes and longer education often have better access to trails, sidewalks and sports facilities. Several of the included studies find that access to facilities is inferior for low-income citizens, but other studies indicate that social and personal factors also play a role in the use of the areas. Adding to this, some evidence possits a lower adoption of for example new bike trails for citizens with shorter education. This leads many of the studies to recommend holistic efforts where improvements in the built environment are initiated simultaneously with other types of efforts that strengthen information, skills and motivation for physical activity. For example, combine cheaper or free access to sports facilities or gyms with marketing and beginners' courses. Another important finding from the equity review is a focus on safety from crime and traffic, which more often is a problem in low income areas, and at the same time a larger perceived barrier for women and elderly people.

**Conclusion:**

The interplay between sociodemographic characteristic and the physical environment is complex, but the review points to some important findings.

